# The Effect of Selenium Supplementation on Amino Acid Accumulation by the Yeast *Saccharomyces cerevisiae* and *Rhodotorula glutinis*

**DOI:** 10.3390/molecules31020254

**Published:** 2026-01-12

**Authors:** Wioletta Sęk, Alicja Synowiec, Katarzyna Pobiega, Marek Kieliszek

**Affiliations:** Department of Food Biotechnology and Microbiology, Institute of Food Sciences, Warsaw University of Life Sciences—SGGW, Nowoursynowska 159 C, 02-776 Warsaw, Poland; wioletta_sek@sggw.edu.pl (W.S.); alicja_synowiec@sggw.edu.pl (A.S.); katarzyna_pobiega@sggw.edu.pl (K.P.)

**Keywords:** selenium, yeast, amino acids, protein, essential amino acid index

## Abstract

Selenium-enriched yeast is considered the most bioavailable dietary form of this trace element. It is already authorised for use in food and feed, making it a key vehicle for closing nutritional Se gaps worldwide. Understanding how selenium accumulation reshapes the amino-acid balance of yeast biomass is crucial for diet-related health benefits and optimising biotechnological processes that rely on high-quality microbial protein. The effect of selenium on yeast amino-acid metabolism is an important area of research due to its potential applications in biotechnology and functional food production. In the presented work, changes in the amino acid profile and protein quality in *Saccharomyces cerevisiae* ATCC 7090 and *Rhodotorula glutinis* CCY 20-2-26 cells grown in the presence of different selenium concentrations (0–40 mg Se^4+^/L) for 24 and 48 h were analyzed. The amino acid content was assessed, and the Chemical Score (CS) and adjusted Essential Amino Acid Index (EAAI) were determined. The results showed that moderate selenium concentrations (2–10 mg Se^4+^/L after 24 h) promoted the accumulation of essential amino acids, such as lysine (53.3 mg/g) and valine (38.0 mg/g) in *S. cerevisiae* and lysine (42.8 mg/g) and valine (41.6 mg/g) in *R. glutinis*. High values of protein quality indices were also obtained under the same conditions—CS exceeding 200% and adjusted EAAI reaching 1.71 for *S. cerevisiae* and 2.05 for *R. glutinis*. It is worth noting, however, that EAAI was presented without methionine and tryptophan. In turn, higher selenium concentrations and longer cultivation time decreased these parameters, especially in the case of *S. cerevisiae*. The obtained data confirm that *R. glutinis* may be a promising source of high-quality protein in selenium-enriched products.

## 1. Introduction

Amino acids play a crucial role in the functioning of living organisms, serving as the fundamental building blocks of proteins and participating in numerous metabolic processes. Among them, essential amino acids (EAA) are particularly important, such as histidine, isoleucine, leucine, lysine, methionine, phenylalanine, threonine, tryptophan, and valine, which must be supplied in the diet because the body is unable to synthesize them [1,2]. In addition to participating in protein synthesis, they play essential roles in gene expression, cell signaling, and nitrogen metabolism. Additionally, it has been demonstrated that branched-chain amino acids (BCAAs) can support cell proliferation and enhance the immune system [3].

Due to their crucial role in cell growth and stress response, the amino acid composition of microorganisms, such as yeast, can directly impact the nutritional quality and biotechnological usefulness of biomass. Some selenium-enriched yeasts, such as *Saccharomyces cerevisiae* CNCM I-3060 and *Yarrowia lipolytica*, have already been approved in the European Union as feed additives or novel foods due to their ability to provide organic forms of selenium with higher bioavailability [4,5]. The European Food Safety Authority (EFSA) has also set a tolerable upper intake level of 255 μg/day for adults, highlighting the importance of controlled selenium supplementation. However, *Rhodotorula glutinis*, despite its proven ability to bioaccumulate selenium, has not yet been approved for use in food or feed applications in the EU [6]. *Rhodotorula glutinis* has gained increasing attention in food and feed biotechnology because it synthesises large quantities of antioxidant carotenoids. As a non-conventional yeast, *R. glutinis* combines carotenoid and lipid production with an advantageous essential amino acid profile, distinguishing it from the classical baker’s yeast *S. cerevisiae*. These unique metabolic traits make *R. glutinis* an attractive candidate for comparative studies on selenium metabolism and its impact on amino acid composition. Selenium (Se) is a trace element essential for the proper functioning of organisms, playing an important role in antioxidant, immunological, and regulatory processes [7,8].

In higher organisms, selenium is incorporated into selenoproteins in the form of selenocysteine, many of which play a crucial role in defense against oxidative stress, such as glutathione peroxidase and thioredoxin reductase [9]. In yeast, selenium at moderate concentrations (2–10 mg Se^4+^/L) can act as a factor that stimulates cellular metabolism and growth. However, at higher concentrations (30–40 mg Se^4+^/L), it becomes toxic, causing the formation of reactive oxygen species (ROS), damage to cell membranes, and disruption of mitochondrial functions [8]. Oxidative stress induced by excess selenium triggers a cellular response that includes both increased activity of detoxification enzymes (such as superoxide dismutases SOD1 and SOD2, catalase CTA1, or glutathione peroxidase GPX2) and changes in the expression of genes regulating redox homeostasis [8,9,10]. In yeast, this can lead to the redistribution of metabolic resources such as amino acids, cellular energy (ATP), and glutathione, which affects the amino acid profile, synthesis of protective proteins, and overall cellular efficiency [8,9].

The yeast *Saccharomyces cerevisiae* is widely used as a model organism in studies on selenium metabolism, as it can convert its inorganic forms, such as selenate and selenite, into organic compounds, including selenomethionine and selenocysteine [11]. Owing to this metabolic capacity, selenium-enriched *S. cerevisiae* is considered one of the most bioavailable sources of dietary selenium. Their cultivation in the presence of Se leads to changes in cellular metabolism, including the amino acid profile, which may affect their nutritional value and potential applications in dietetics and the food industry [8]. Previous studies have shown that environmental factors, including oxygen availability and the presence of metals, can alter the amino acid profile by modulating central metabolic pathways [12]. Such changes may alter the pool of amino acid precursors and the activity of enzymes responsible for their synthesis, resulting in variations in the cellular amino acid composition [12]. Amino acid metabolism in yeast is a complex network of interactions involving both catabolic and anabolic pathways, and the regulation of their content in the cell depends on the availability of nutrients and the presence of stress factors [8]. However, little is known about how selenium supplementation explicitly affects yeast’s amino acid composition and protein quality. Comparative studies between different yeast species are also lacking, despite their potential importance for biotechnology and nutrition.

Therefore, the objective of this study was to investigate how different selenium concentrations (0–40 mg Se^4+^/L) and cultivation times (24–48 h) affect the amino acid composition and protein quality of *Saccharomyces cerevisiae* ATCC 7090 and *Rhodotorula glutinis* CCY 20-2-26. To the best of our knowledge, no previous study has comprehensively quantified how graded Se(IV) supplementation reshapes the complete amino acid profile and protein quality parameters (CS and adjusted EAAI) in a conventional yeast (*S. cerevisiae*) compared with a non-conventional yeast (*R. glutinis*) under identical cultivation conditions. By studying these two species in parallel, we aimed to highlight standard mechanisms of selenium metabolism and species-specific responses, thereby providing new insights into the metabolism of amino acids in selenium-enriched yeast and their potential applications as functional food ingredients and dietary supplements.

## 2. Results and Discussion

### 2.1. The Effect of Selenium on the Amino Acid Profile of the Yeast Saccharomyces cerevisiae ATCC 7090

Across 0–40 mg Se^4+^/L and 24–48 h, we observed condition-dependent shifts in the cellular amino acid pool of *S. cerevisiae*. Table 1 summarizes these shifts and highlights time and dose-dependent patterns. The analysis revealed significant changes in the amounts of the tested compounds, which were dependent on both their concentration and cultivation time. The data presented in Table 1 confirm that a longer cultivation time led to a general reduction in the content of most amino acids, which may result from their metabolism or degradation in the later phases of yeast cell growth.

Among the amino acids analyzed, glutamic acid showed the highest content in yeast biomass under all conditions, reaching a maximum value of 93.7 mg/g after 24 h of culture with the addition of 10 mg/L of selenium. Aspartic acid and lysine were also amino acids with relatively high concentrations (62.2 and 44.4 mg/g for the control sample) after 24 h. In the yeast biomass obtained after cultivation in a medium enriched with 10 mg Se^4+^/L, the content of the same amino acids increased accordingly to 60.2 and 53.3 mg/g, respectively, indicating their intensive synthesis or accumulation in yeast cells. It should be noted, however, that the differences obtained for these amino acids between the control sample and the sample enriched with selenium (10 mg Se^4+^/L) were not statistically significant. The lowest amounts were recorded for cysteine, the content of which remained low regardless of the culture conditions; its values ranged between 3.6 mg/g and 5.7 mg/g, which suggests limited synthesis or rapid catabolism of this amino acid in the studied system. The contents obtained were statistically different.

The addition of selenium significantly affected the content of amino acids, with an increase in their level observed in many cases at lower selenium concentrations (2–10 mg Se^4+^/L), while higher doses (20–40 mg Se^4+^/L) resulted in a downward trend. Significant changes were observed in amino acids such as glutamic acid, aspartic acid, lysine, and tyrosine, which showed the most pronounced differences in response to varying selenium concentrations and culture times. Glutamic acid, glycine, and lysine reached maximum levels at 10 mg/L after 24 h (93.7, 40.6, and 53.3 mg/g, respectively), but decreased markedly at 40 mg/L and after 48 h (to 48.1, 27.4, and 34.5 mg/g). It is worth noting that the selenium concentration used (0–20 mg Se^4+^/L) did not significantly affect the content of branched-chain amino acids (valine, leucine, isoleucine) after 48 h.

Culture time had an apparent effect on the amino acid profile. After 48 h, a general reduction in the content of most amino acids was noted compared to 24 h, suggesting their intensive use by cells in the later phases of growth. A particularly pronounced decrease after a more extended cultivation period was observed in the case of threonine, the content of which decreased from 27.6 mg/g (0 mg Se^4+^/L, 24 h) to 16.3 mg/g (40 mg Se^4+^/L, 24 h), and serine—from 25.5 mg/g (0 mg Se^4+^/L, 24 h) to 17.6 mg/g (40 mg Se^4+^/L, 24 h), which may indicate their more intensive degradation or increased consumption in metabolic processes. It is worth emphasizing that the results obtained for threonine were statistically significant. In the case of tyrosine, a decrease in its content was observed after 48 h at a concentration of 10 mg/L of selenium (from 13.8 to 10.9 mg/g), which may suggest increased metabolism or delayed synthesis in response to this element. However, it is worth noting that the obtained results were not significant.

**Figure 1:**
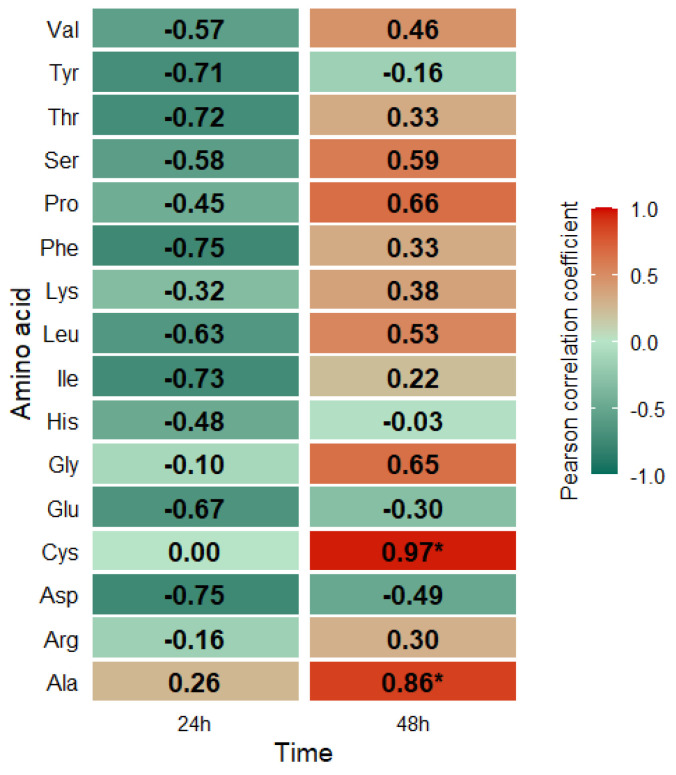
Heatmap of Pearson correlations between amino acid concentrations in *S. cerevisiae* biomass and selenium content over time. Asterisks indicate statistically significant correlations (*p*-value < 0.05).

Correlation analysis (Figure 1) between amino acid concentrations in *S. cerevisiae* biomass and selenium content over time revealed diverse relationships that may indicate specific metabolic mechanisms regulated by selenium. These include oxidative stress and genes regulating amino acid metabolism.

The color gradient in the figure represents correlation coefficients, with shades of red indicating positive correlations and shades of green indicating negative correlations. Based on the obtained results, it was noted that for specific amino acids, including glycine, cysteine, alanine, proline, and glutamic acid, a positive correlation exists with culture time, suggesting their accumulation or enhanced biosynthesis during later growth phases. Branched-chain amino acids, such as valine, leucine, and isoleucine, tend to have a negative correlation, which may indicate their intensive metabolic consumption or limited synthesis during more extended culture periods. Cysteine exhibits a strong negative correlation after 24 h yeast culture, which may indicate its susceptibility to degradation or utilization in various metabolic pathways, such as glutathione synthesis. The observed correlations confirm that the effect of selenium on amino acid metabolism is dynamic and dependent on both the composition of the culture medium and the duration of cell exposure to this element.

The results indicated that selenium can affect amino acid metabolism in *S. cerevisiae* depending on its concentration and yeast growth phase. The identified trends enable further studies of the mechanisms regulating these processes and the assessment of the potential applications of selenium in fermentation biotechnology.

To more fully interpret the obtained results, they were compared with data presented in the available literature. Such a comparison enables us to assess the consistency of the observed relationships with previous studies and to identify potential differences resulting from the applied experimental conditions. Literature data presented by Kieliszek et al. [13] showed that selenium supplementation (20 mg Se^4+^/L) led to an increase in the total amino acid content by 12% in *C. utilis* (from 510 mg/g to 571 mg/g) and by 5% in *S. cerevisiae* (from 485 mg/g to 509 mg/g). At the same time, an increase in the level of glutamic acid in *S. cerevisiae* by 5 mg/g (from 73 mg/g to 78 mg/g) was observed, and lysine by 8 mg/g (from 44 mg/g to 52 mg/g). Similar relationships were observed in our study involving the *S. cerevisiae* ATCC 7090 strain, where glutamic acid reached a maximum value of 93.7 mg/g after 24 h of culture with the addition of 10 mg Se^4+^/L of selenium, compared to 73.6 mg/g in the control sample. Lysine in these conditions increased to 53.3 mg/g, which is a value similar to the literature data. Lysine in these conditions increased to 53.3 mg/g, which is a value similar to the literature data [13]. However, with a longer cultivation time (48 h), its content decreased drastically by more than 15.9 mg/g—a phenomenon not observed in the studies by Kieliszek et al. [13]. Proline in *C. utilis* remained at a stable level of about 20 mg/g regardless of selenium supplementation. In comparison, in *S. cerevisiae*, it was shown to decrease from 22 mg/g to 17.7 mg/g. An analogous trend was confirmed in this study, where the proline level in *S. cerevisiae* ATCC 7090 decreased from 20.6 mg/g (control conditions) to 17.0 mg/g (40 mg Se^4+^/L, 24 h). These results may indicate an increased use of proline in the mechanisms of cell protection against oxidative stress or a limited activity of the enzymes involved in the biosynthesis of this amino acid. In the case of glycine, Kieliszek et al. [13] noted an increase from 28 mg/g to 36 mg/g in *C. utilis* and from 26 mg/g to 30 mg/g in *S. cerevisiae*. Even more pronounced changes were noted in the conducted analyses: the glycine content in *S. cerevisiae* ATCC 7090 increased after 24 h of culture with 10 mg Se^4+^/L, gradually decreasing at higher doses. This suggests that moderate selenium concentrations may support glycine biosynthesis, whereas excess selenium leads to an inhibitory effect.

In summary, the total amino acid content was increased in response to selenium supplementation, as shown in the literature data and our studies. However, in the case of the *S. cerevisiae* ATCC 7090 strain, some values, especially for glutamic acid and glycine, were higher than those reported in the literature, and the dynamics of changes in lysine and proline content indicated more substantial adaptive effects of the yeast to stress conditions induced by the presence of selenium.

Other metals, such as copper and manganese, have also been shown to influence amino acid metabolism through mechanisms related to oxidative stress and redox imbalance [14,15]. In yeast, copper can activate protective pathways regulated by Yap1p and detoxification enzymes such as glutathione peroxidase and thioredoxin reductase, while accumulation of AICAR may disrupt purine and amino acid synthesis [16]. These findings suggest that the reduction in essential amino acids observed at higher selenium concentrations may be part of a broader stress response mechanism common to multiple elements.

### 2.2. Analysis of CS and Adjusted EAAI Indices in Yeast S. cerevisiae ATCC 7090

To assess protein quality in *Saccharomyces cerevisiae* cells, values of the Chemical Score (CS) for selected essential amino acids and the Essential Amino Acid Index (EAAI) were calculated. CS is defined as the ratio of the concentration of a given crucial amino acid in the sample to its concentration in the WHO/FAO reference pattern, with the lowest resulting value indicating the limiting amino acid for protein quality. EAAI represents the geometric mean of the ratios of all essential amino acids in the sample to their corresponding values in the reference pattern, reflecting the overall biological value of the protein relative to WHO/FAO standards [17,18].

**Figure 2:**
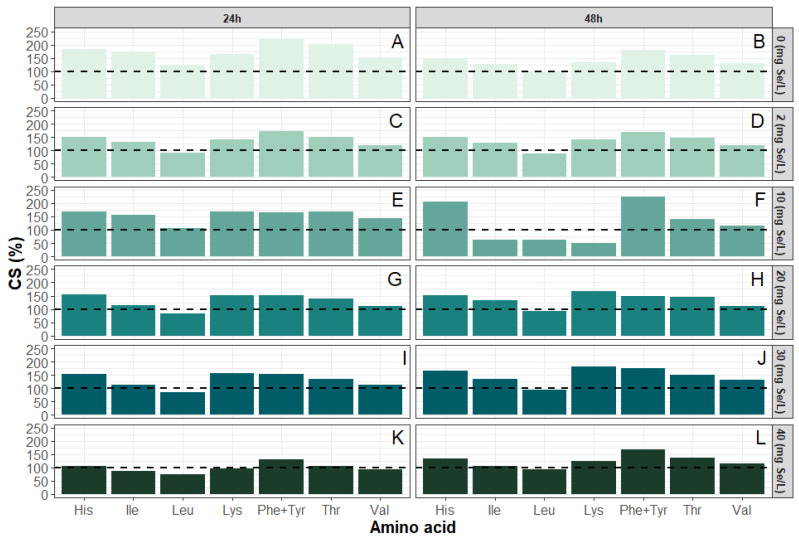
Chemical score (CS) for *S. cerevisiae* yeast cultivated with selenium addition for 24 and 48 h ((**A**): 0 mg Se^4+^/L, (**C**): 2 mg Se^4+^/L, (**E**): 10 mg Se^4+^/L, (**G**): 20 mg Se^4+^/L, (**I**): 30 mg Se^4+^/L, (**K**): 40 mg Se^4+^/L) and 48 h ((**B**): 0 mg Se^4+^/L, (**D**): 2 mg Se^4+^/L, (**F**): 10 mg Se^4+^/L, (**H**): 20 mg Se^4+^/L, (**J**): 30 mg Se^4+^/L, (**L**): 40 mg Se^4+^/L).

In the analyzed samples, the highest CS values (Figure 2) were obtained for the sum of phenylalanine and tyrosine, which reached 223.5% after 48 h of culture with the addition of 10 mg/L selenium and 220.0% in the control sample after 24 h. High values were also obtained for threonine, the CS of which was 201.1% for the control sample cultured for 24 h and 167.1% for the sample after 24 h of culture in 10 mg/L selenium. In the case of histidine, the maximum CS value reached 204.42% for yeast cultured with 10 mg Se^4+^/L for 48 h. For isoleucine, the maximum CS value was 175.25% in the control sample without selenium addition after 24 h. Such values indicate excellent availability of these amino acids in yeast biomass, especially under control conditions and at moderate selenium concentrations. A different trend was observed for lysine and leucine, which in some samples reached values significantly below 100%, indicating their potential role as limiting amino acids.

**Figure 3:**
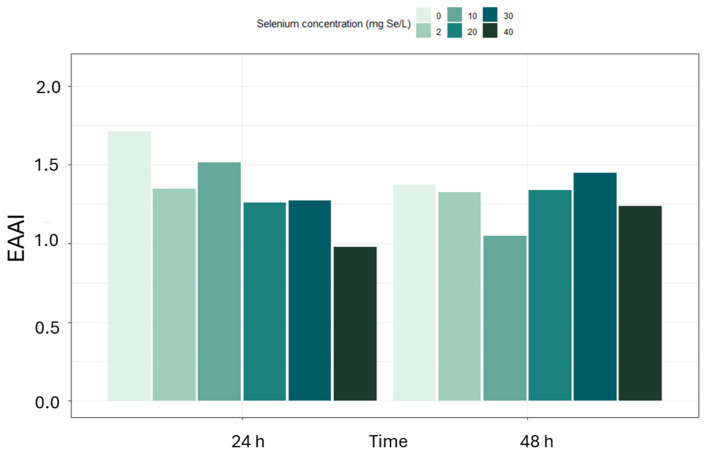
Essential amino acid index (adjusted EAAI) for *S. cerevisiae* yeast cultivated with selenium addition (0–40 mg Se^4+^/L) for 24 and 48 h.

The lowest CS values for leucine and lysine were recorded in the culture with 10 mg/L of selenium added for 48 h, where they were 62.1% and 48.9%, respectively, as well as in the sample grown in the presence of 40 mg/L of selenium for 24 h (leucine—74.6%, lysine—96.7%). A predominant decrease in CS values was observed at higher selenium concentrations and after 48 h of culture, suggesting that most essential amino acids are susceptible to the metabolic stress imposed by selenium, even though a few residues (e.g., Phe + Tyr at 30 mg Se^4+^/L) exhibit the opposite behavior. The adjusted EAAI index ranged from 0.98 to 1.71. EAAI values exhibited a complex, dose and time-dependent response to selenium (Figure 3): in the control culture, adjusted EAAI decreased from 1.71 at 24 h to 1.37 at 48 h, with the lowest value (1.05) recorded at 48 h under 10 mg Se^4+^/L. At higher selenium concentrations (20–40 mg Se^4+^/L), adjusted EAAI partially increased to 1.34–1.45 after 48 h. This non-monotonic behavior suggests an initial disturbance in amino acid balance followed by adaptive metabolic shifts in response to more intense selenium stress. In the case of the control sample without selenium supplementation, after 48 h, the adjusted EAAI was 1.37, whereas with 2 mg/L of selenium after 24 h, it was 1.35. It is worth noting that even in samples with moderate selenium concentrations, such as 30 mg Se^4+^/L cultured for 48 h, the adjusted EAAI value was still relatively high at 1.45, which may indicate a beneficial effect of shorter exposure or a tolerable range of the element’s action. The lowest EAAI values were recorded in the culture with 40 mg/L of selenium supplementation for 24 h (0.98) and in the culture with 10 mg/L of selenium supplementation for 48 h (1.05). Such a low level may indicate significant deficiencies of limiting amino acids, which is confirmed by low CS values for leucine and lysine.

Studies conducted by Kieliszek et al. [19] provide valuable information on the effect of selenium on the proteome of yeast *S. cerevisiae* ATCC MYA-2200. In conditions of selenium supplementation (20 mg Se^4+^/L), changes in the expression level of many proteins related to cellular metabolism were observed, including mitochondrial malate dehydrogenase (MDH), ATP-dependent RNA helicase (dbp3), and tryptophan synthase (Trp-DMAT). An increase in the level of MDH, a protein involved in the TCA cycle and metabolite transport in mitochondria, was particularly significant, suggesting an increased demand for energy and cell adaptation to stress induced by the presence of selenium. An increase in the AlaX protein, responsible for the correct translation of amino acids, was also observed, which may indicate that cells are protected against translation errors under oxidative stress. The decrease in glyceraldehyde-3-phosphate dehydrogenase (GAPDH) suggested the inhibition of glycolysis under selenium stress conditions. These results confirm that selenium induces extensive changes in the metabolism of amino acids and yeast proteins, which may lead to modifications of protein quality indicators such as adjusted EAAI or CS. However, their direct numerical values were not presented in the study. The described changes indicate an attempt to maintain cellular homeostasis through increased expression of adaptive and detoxifying proteins such as glutathione peroxidase (GPx), thioredoxin reductase (TrxR), and glutathione synthase, which play a key role in neutralizing reactive oxygen species (ROS) and maintaining redox balance [20,21].

In the study by Agboola et al. [22], it was shown that yeasts of different species (including *Saccharomyces cerevisiae*, *Cyberlindnera jadinii*, *Kluyveromyces marxianus*, *Blastobotrys adeninivorans*, and *Wickerhamomyces anomalus*) are characterized by high protein content (38–55% of dry matter) and a favorable amino acid profile, similar to that of fish protein and soy protein. The most significant amounts in the amino acid composition of yeast were glutamic acid (10.8–13.3 g/16 g N) and aspartic acid (7.1–10.1 g/16 g N). However, the analysis indicated limitations in the content of some essential amino acids, particularly methionine, cysteine, lysine, and phenylalanine. In the biomass of *S. cerevisiae* yeast, the methionine content was 1.8 g/16 g N, while in *C. jadinii* it was only 1.1 g/16 g N. The EAAI index, which assesses overall protein quality, was lower for yeast than for fishmeal (which, as a reference protein, is considered ideal for feeding Atlantic salmon and rainbow trout, and has an EAAI of 100). *Wickerhamomyces anomalus* showed the highest EAAI value among the analyzed yeasts (79), while *S. cerevisiae* showed 76, and the lowest value was demonstrated by *B. adeninivorans* yeast (67). A high glutamate content in yeast may enhance nitrogen metabolism and promote cell growth under aerobic conditions. This phenomenon is also confirmed by the results of this study, which show an increase in glutamic acid levels with moderate selenium supplementation.

In the studies conducted by Pereira et al. [23], the effect of different yeast strains from the *Saccharomyces* genus on the amino acid content in wines from the Arinto variety was assessed. Among the four strains used (*S. bayanus* EC1118, *S. cerevisiae* CY3079, *S. bayanus* QA23, and native yeasts), the highest total content of amino acids was obtained in wines fermented with the CY3079 strain, reaching 470.74 mg/L. The lowest content was recorded for the EC1118 strain (343.06 mg/L). All fermentations showed proline dominance, ranging from 97.08 mg/L (EC1118) to 157.25 mg/L (CY3079). The next most abundant amino acids were alanine (30.49–55.36 mg/L), leucine (26.72–31.24 mg/L), lysine (24.97–29.97 mg/L), and arginine (23.63–31.39 mg/L). The content of the remaining amino acids was lower and usually did not exceed 22 mg/L. It is worth noting that fermentation with native yeasts resulted in a higher share of esters in the volatile aromatic compounds profile than fermentation with commercial strains, which may suggest a different nitrogen and amino acid metabolism pathway under these conditions [23].

Sirisena et al. [24] studied the effect of aerobic and anaerobic conditions on amino acid metabolism in yeast *S. cerevisiae* ATCC 5574. The analysis revealed significant differences in their profiles—glutamic acid and histidine were present in significantly higher concentrations under aerobic conditions (229.78 nmol/mg and 66.87 nmol/mg, respectively) compared to anaerobic conditions (12.45 nmol/mg and 0.65 nmol/mg, respectively). In turn, lysine, alanine, and proline were more abundant in cells grown anaerobically, which suggests that growth conditions determine the metabolic pathways responsible for amino acid synthesis and accumulation.

Compared to our earlier results, we also observed variability in amino acid content depending on the culture conditions; however, the differences were not as extreme. Glutamic acid in *S. cerevisiae* showed a maximum value of 93.7 mg/g after 24 h in the presence of 10 mg/L selenium, which is much higher than that of yeast grown in anaerobic conditions. Still, it did not reach a high ratio as in the cited studies. Literature data confirm that glutamic acid, aspartic acid, and alanine are key elements of the pool of free amino acids in yeast cells, and their biosynthesis is strongly related to the activity of the TCA cycle, especially in aerobic conditions, which was described, among others, by Sirisena et al. [24]. In aerobic conditions, their production is increased, leading to the intensification of protein synthesis, as also observed in the results of this study, where glutamic acid reached its highest concentrations at moderate selenium concentrations. The increase in alanine content noted in the literature under anaerobic conditions was not as pronounced in the analyses conducted, which may be due to different regulations of nitrogen metabolism depending on the yeast species [24].

In further analysis, the CS index values were compared to the results published by other authors, including studies on brewing yeast. In the studies by Podpora et al. [25], the nutritional value of yeast extracts from brewing yeast was assessed, analyzing, among others, the profile of essential amino acids based on the Chemical Score (CS). The authors noted high CS values for valine (154%), isoleucine (157%), threonine (121%), and lysine (114%) in the extract, which indicated good protein quality.

In summary, moderate selenium concentrations (up to 10 mg/L) and a shorter incubation time (24 h) favored the attainment of a more balanced profile of essential amino acids and a higher biological value of protein. High selenium concentrations and more prolonged exposure led to the deterioration of these parameters, mainly by limiting the content of lysine and leucine. Detailed analysis showed that CS of lysine and leucine decreased significantly with increasing selenium concentration, while histidine and threonine reached the highest values under conditions of moderate supplementation. Notably, phenylalanine and tyrosine content remained at a high level regardless of the culture conditions used, indicating the high stability of these amino acids in response to selenium stress.

### 2.3. The Effect of Selenium on the Amino Acid Profile of the Yeast Rhodotorula glutinis CCY 20-2-26

In *R. glutinis*, selenium also modulated the amino acid profile in a time- and dose-dependent manner. Table 2 visualizes these responses and their dependence on selenium concentration and cultivation time.

Similarly to *S. cerevisiae*, glutamic acid had the highest content of all amino acids. Its maximum value (102.8 mg/g) was recorded after 48 h of cultivation in the presence of 2 mg/L of selenium. At the same time, at higher concentrations, the content of this amino acid was lower and statistically significant. High values were also obtained for aspartic acid, arginine, and glycine. Aspartic acid content reached a maximum of 63.7 mg/g after 48 h of culture in the presence of 20 mg/L of selenium. At the same time, arginine showed the highest concentration at 63.4 mg/g in the control sample after 24 h. Glycine reached the highest content (64.5 mg/g) after 48 h of culture with the addition of 20 mg/L of selenium. The obtained result was statistically significant in relation to the glycine content obtained from yeast biomass grown in media with the remaining selenium doses (30–40 mg Se^4+^/L). The lowest concentrations were recorded for cysteine, the content of which remained low regardless of the selenium concentration and culture time—it ranged from 3.2 mg/g (20 mg Se^4+^/L, 24 h) to 4.9 mg/g (40 mg Se^4+^/L, 24 h), which may suggest a limited synthesis of this amino acid or its rapid catabolism. The presented results did differ statistically. The addition of selenium affected individual amino acids in different ways.

In most cases, moderate doses of selenium (2–10 mg/L) increased the amino acid content in cells, while higher concentrations of this element (20–40 mg Se^4+^/L) resulted in their decrease. This was particularly visible in the case of glutamic acid, glycine, and alanine, the maximum values of which were recorded under conditions of medium selenium concentration. At the same time, their content gradually decreased with increasing concentration of this element. Alanine reached its highest level (59.0 mg/g) after 48 h in the presence of 20 mg/L of selenium; however, in a longer culture and at higher concentrations, its level decreased. Branched-chain amino acids increased at 10–20 mg Se^4+^/L, but decreased at 30–40 mg Se^4+^/L.

The effect of culture time on the amino acid content was significant. After 48 h, in most cases, a decrease in the number of amino acids was observed compared to 24 h, which may indicate their intensive use by cells in later growth phases or their increased degradation. In the case of aspartic acid and serine, such a marked decrease in content was not noted for the other amino acids, especially after 48 h of culture in higher selenium concentrations, which may suggest their essential role in cell adaptation to stress conditions. Lysine, phenylalanine, and histidine exhibited distinct responses to selenium, with the highest values of these amino acids observed at concentrations ranging from 10 to 20 mg Se^4+^/L. It is worth noting that lysine in *R. glutinis* behaved differently than in S. cerevisiae, where its content was significantly reduced at higher selenium concentrations.

**Figure 4:**
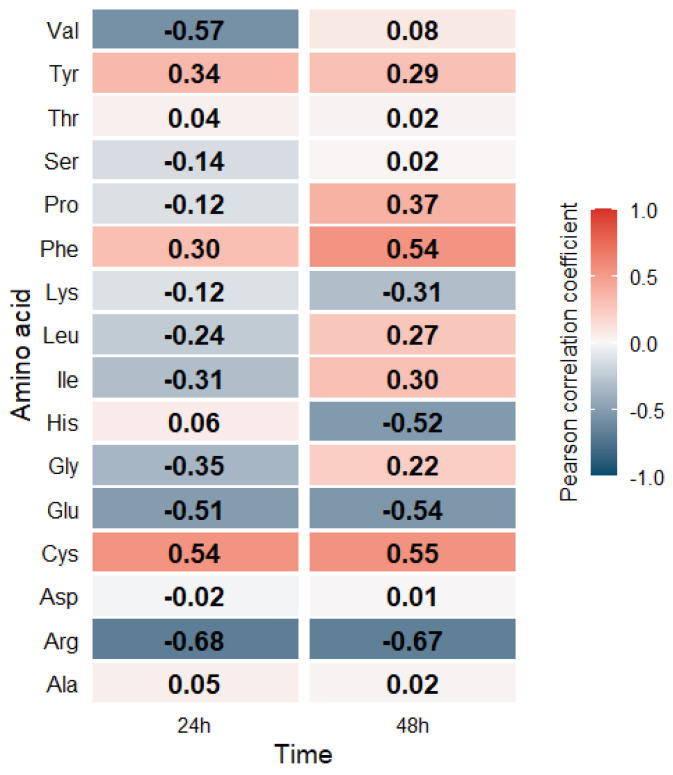
Heatmap of Pearson correlations between amino acid concentrations in *R. glutinis* biomass and selenium content over time.

The correlation analysis between amino acid concentrations in *R. glutinis* biomass and selenium content over time revealed diverse depending on the group of compounds studied (Figure 4). In contrast to *S. cerevisiae*, a more varied range of correlation values was observed for this strain, including both positive and negative relationships. In this figure, the color gradient represents correlation coefficients, with red shades indicating positive correlations and blue shades indicating negative correlations.

Amino acids such as glutamic acid, arginine, and lysine, for example, showed a negative correlation with culture time, which may suggest their intensive consumption in the later stages of cell growth. In particular, glutamic acid, which reached its highest values in the initial culture period, showed a significant decrease after 48 h, which may be due to its role as a key metabolite in the biosynthesis of proteins and other nitrogenous compounds. A similar trend was observed for lysine, which showed a strong negative correlation with culture time, suggesting that more prolonged cell exposure to selenium may lead to its more intensive degradation or metabolic utilization.

Cysteine, on the other hand, showed a positive correlation, which may indicate its accumulation in cells later in the culture period. This relationship may result from cysteine’s role in protecting cells from oxidative stress, particularly in the presence of selenium, which can impact redox homeostasis and sulfur metabolism. A similar pattern of correlation was observed after 48 h of culture for proline, leucine, and phenylalanine, suggesting that these amino acids may play specific protective or adaptive roles in response to changing culture conditions.

Although both *S. cerevisiae* and *R. glutinis* exhibit increases in key essential amino acids at moderate selenium concentrations (2–10 mg Se^4+^/L, 24 h) for example, lysine at approximately 53.3 mg/g in *S. cerevisiae* versus 42.8 mg/g in *R. glutinis*, and valine at 31.1 mg/g versus 30.8 mg/g at higher concentrations (20–40 mg Se^4+^/L, 24 h) the decline in amino acid content is substantially deeper and more uniform in *S. cerevisiae*, whereas in *R. glutinis* it is milder and more heterogeneous. The adjusted EAAI for *S. cerevisiae*, initially ranging from 0.98 to 1.71, falls below 1.0 at 40 mg Se^4+^/L. At the same time, *R. glutinis* maintains consistently high values (1.37–2.05) and even at the highest selenium doses retains an adjusted EAAI of 1.42–1.50. Moreover, the Chemical Score for phenylalanine + tyrosine reaches 281% in *R. glutinis* compared to a maximum of 223% in *S. cerevisiae*, indicating superior protein quality.

These differences have important practical implications: thanks to its high and stable protein quality as well as its additional capacity to produce carotenoids and accumulate lipids [26] and to generate industrially applicable enzymes such as lipases, invertases, and pectinases [27], *R. glutinis* is a desirable candidate for applications in selenium and protein-fortified foods, food colorants and dietary supplements, animal (livestock and aquaculture) feeds [28], and biotechnology (EPS production) [29], whereas S. cerevisiae, although valuable as a source of selenomethionine under moderate supplementation [30], loses its utility at higher selenium doses.

Similar effects have been reported for copper, where His, Lys, and Glu levels reduced with increasing Cu^2+^ concentration, while Pro and Thr were less affected [15]. Histidine, in particular, showed strong sensitivity to both copper and selenium, possibly due to AICAR accumulation disrupting purine and amino acid biosynthesis pathways [16]. These parallels suggest that selenium and copper may trigger overlapping stress responses in yeast. In this study, under control conditions, the histidine level was 16.26 mg/g. At a selenium concentration of 40 mg/L, it dropped to 11.28 mg/g, representing a reduction of approximately 30.6%. Both elements, therefore, hurt the accumulation of histidine, which may result from their impact on the enzymes responsible for its biosynthesis.

Glutamic acid, on the other hand, showed different reactions to the presence of copper and selenium. Experiments conducted by Dimova et al. [15] demonstrated that low concentrations of Cu^2+^ stimulated an increase in glutamic acid content in yeast cells, whereas higher concentrations led to a decrease. The studies showed that glutamic acid increased under moderate selenium concentrations (up to 93.7 mg/g at 10 mg Se^4+^/L). Still, at higher doses (40 mg Se^4+^/L), its level decreased to 42.8 mg/g. A similar compensatory effect was observed for lysine, they indicated that the level of this amino acid gradually decreased with increasing Cu^2+^ concentration. In contrast, our study, reached a maximum at a concentration of 10 mg Se^4+^/L (53.3 mg/g), after which, with a further increase in selenium concentration to 40 mg/L, its level dropped to 31.5 mg/g. This means that both copper and selenium can stimulate the metabolism of these amino acids under certain conditions, while their excess leads to an inhibiting effect.

Opposite relationships were observed for proline. In the publication [15], the authors emphasized that the content of this amino acid remained relatively stable even at high copper concentrations. At the same time, in the experiment, the proline level systematically decreased with increasing selenium concentration (from 20.6 mg/g to 17.2 mg/g). This may indicate that the regulatory mechanisms associated with proline accumulation are less susceptible to copper, while in the case of selenium, proline is more intensively used in detoxification processes.

Proline protects cells from oxidative stress, acts as a scavenger of reactive oxygen species (ROS), and stabilizes protein structures and cell membranes. Moreover, its catabolism leads to the formation of NADPH, which is essential in the regeneration processes of glutathione the primary cellular antioxidant that supports the maintenance of redox homeostasis and effective neutralization of free radicals [31,32]. According to the literature, proline plays an essential protective role in oxidative stress conditions, acting as a scavenger of reactive oxygen species (ROS) and a stabilizer of protein structures and cell membranes [31]. High levels of oxidative stress caused by excess selenium may increase the demand of cells for proline to protect against oxidative damage, which leads to its more intensive consumption and reduced intracellular levels [33].

Liang and Zhou [14] demonstrated that Cu^2+^ and Mn^2+^ affect amino acid metabolism in yeast through different mechanisms: copper induces oxidative stress and mitochondrial dysfunction, whereas manganese does not generate ROS. Similarly to copper, selenium at high concentrations triggered oxidative stress in our study, as reflected in glutamic acid dynamics (an increase at 10 mg Se^4+^/L and a decrease at 40 mg Se^4+^/L), consistent with the patterns reported for Cu^2+^ [14].

Lysine in the yeast *S. cerevisiae* BY4742 reached a maximum concentration of 47 mg/g at 50 mg/L Cu^2+^ and then decreased to 25 mg/g at 150 mg Cu^2+^/L. In our research, analyses show that the lysine content increased to 53.3 mg/g at 10 mg Se^4+^/L and then decreased to 31.5 mg/g at 40 mg Se^4+^/L, which similar to copper, may result from the induction of oxidative stress, which leads to changes in the activity of enzymes involved in protein biosynthesis and degradation. As shown by Liang and Zhou [14], high concentrations of Cu^2+^ disrupt mitochondrial function and increase the level of reactive oxygen species (ROS), which may affect the expression of genes encoding enzymes responsible for amino acid metabolism, including lysine. Such enzymes include, among others, branched-chain α-ketoacid dehydrogenase (BCKDH), involved in the catabolism of branched-chain amino acids, and diaminopimelate synthase (DAP synthase), crucial for lysine biosynthesis in the diaminopimelate pathway [31,32].

An opposite trend was observed for proline, the level of which remained stable (~20 mg/g) in the presence of Cu^2+^ in the studies by Liang and Zhou [14], while in the studies on selenium, it decreased from 20.6 mg/g (0 mg Se^4+^) to 17.2 mg/g (40 mg Se^4+^/L). This may suggest that Se^4+^ more intensively involves proline in detoxification mechanisms than Cu^2+^, probably due to the greater production of reactive oxygen species (ROS), which require neutralization by specific amino acids, such as proline, cysteine, methionine, and histidine. Proline protects cells from oxidative stress, by acting as a free radical scavenger and stabilizing of protein structures [31]. Cysteine and methionine, due to the presence of thiol groups, directly neutralize ROS, and their residues are also involved in the regeneration of glutathione, one of the most important cellular antioxidants [32]. In turn, due to an imidazole group, histidine binds transition metal cations and exhibits antioxidant properties [21]. In summary, both selenium and copper affect amino acid metabolism through oxidative stress, which can lead to the modification of the activity of enzymes involved in amino acid biosynthesis and degradation, as well as the redistribution of cellular resources towards protective mechanisms. Manganese, on the other hand, acts differently—it does not generate ROS significantly, resulting in a different profile of changes in the level of amino acids [14].

In summary, both Cu^2+^ and Se^4+^ ions affect amino acid metabolism in *S. cerevisiae*, but their mechanisms of action are different. Copper exerts its toxic effects mainly by inducing oxidative stress and mitochondrial dysfunction, which leads to disruptions in protein biosynthesis, as shown in the studies of Liang and Zhou [14] and Dimova et al. [15]. In turn, depending on the concentration, selenium can exhibit both stimulating and toxic effects. Moderate concentrations of Se^4+^ (2–10 mg/L) enhance the protein’s amino acid profile and biological value by activating antioxidant pathways and regulating nitrogen metabolism. On the other hand, high concentrations of selenium (30–40 mg/L) lead to the overproduction of reactive oxygen species (ROS), mitochondrial dysfunction, and a redistribution of metabolic resources towards detoxification processes, resulting results in a decrease in the content of key amino acids, such as leucine and lysine. This mechanism of selenium action was also confirmed in the present study, where an apparent decrease in the content of essential amino acids was observed in conditions of higher concentrations of this element. The survey conducted by Batista et al. [34] showed that supplementation of *S. cerevisiae* BY4741 with sodium selenite (1 mmol/L) caused significant changes in the amino acid profiles of yeast cells. The glutamic acid content increased from 5.29 nmol/mg to 11.14 nmol/mg of protein, confirming its key role in detoxification mechanisms and response to oxidative stress.

In the study, an increase in glutamic acid was also observed in *S. cerevisiae* ATCC 7090 at moderate selenium concentrations (93.7 mg/g at 10 mg Se^4+^/L), which may indicate similar protective mechanisms. Similarly, the proline level increased from 2.66 nmol/mg to 7.15 nmol/mg protein in response to selenium. This result is consistent with the trend observed in the discussed work, where the decreasing proline level under stress conditions suggested its intensive use in protective mechanisms against ROS. Alanine, a vital energy amino acid, also increased, from 5.53 nmol/mg to 7.64 nmol/mg protein after exposure to selenium. This is consistent with observations indicating cell adaptation towards increased production of energy precursors.

On the other hand, the content of serine decreased from 6.21 nmol/mg to 4.33 nmol/mg, and aspartic acid from 3.72 nmol/mg to 2.44 nmol/mg. This decrease may indicate a shift in metabolic pathways from amino acid biosynthesis to pathways related to the response to oxidative stress. The level of methionine decreased significantly from 0.35 nmol/mg to 0.19 nmol/mg of protein and tyrosine from 1.57 nmol/mg to 1.00 nmol/mg after selenium supplementation. This finding is consistent with the results obtained in the conducted study, which also noted a decrease in the levels of selected amino acids under higher selenium concentrations. It was also observed that the combination of selenium and gamma radiation led to a further increase in the level of serine (to 7.38 nmol/mg) and glycine (from 2.55 nmol/mg to 5.73 nmol/mg of protein), which indicates a strong activation of the pathways of one-carbon metabolism, such as the folate cycle and the methionine cycle. These pathways play a crucial role in nucleotide synthesis, DNA methylation, and the regulation of redox balance, which may be a cellular response to oxidative stress induced by selenium and radiation [20,21]. The studies by Batista et al. [34] confirm that selenium induces adaptive changes in amino acid metabolism in yeast by increasing the synthesis of glutamate, proline, and alanine and reducing the levels of serine, aspartic acid, and methionine, similar to what was observed in our work.

The results of the correlation analysis confirm that amino acid metabolism in *R. glutinis* is dynamic and dependent on culture time. In contrast to *S. cerevisiae*, where decreases in amino acid content with cultivation time were dominant, in the case of *R. glutinis*, some amino acids tend to accumulate over a more extended period. This may be due to differences in metabolic strategies between these two yeasts and the effects of selenium on the regulation of amino acid biosynthesis. Further studies will enable a more precise determination of the mechanisms underlying these processes and their potential applications in biotechnology.

Heatmap analysis (Figure 4) confirms that the effect of selenium on amino acid metabolism in *R. glutinis* is complex and may depend on the specificity of yeast metabolism and the availability of other nutrients in the medium. The results indicated that moderate selenium concentrations may promote the accumulation of some amino acids. At the same time, higher doses lead to their reduction, probably due to increased metabolic consumption or changes in the activity of enzymes responsible for their synthesis. Further studies on the mechanisms of these changes will allow for a better understanding of the effect of selenium on amino acid metabolism in *R. glutinis* and the potential applications of this element in fermentation biotechnology.

### 2.4. Analysis of CS and Adjusted EAAI Indices in the Yeast Rhodotorula glutinis CCY 20-2-26

**Figure 5:**
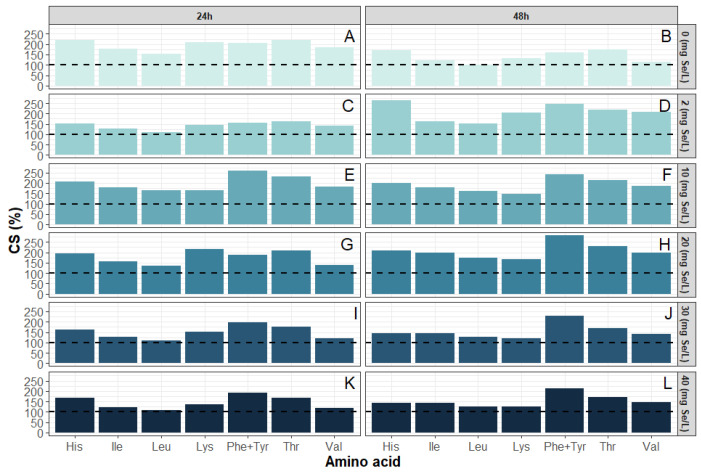
Chemical score (CS) for *R. glutinis* yeast cultivated with selenium addition for 24 and 48 h ((**A**): 0 mg Se^4+^/L, (**C**): 2 mg Se^4+^/L, (**E**): 10 mg Se^4+^/L, (**G**): 20 mg Se^4+^/L, (**I**): 30 mg Se^4+^/L, (**K**): 40 mg Se^4+^/L) and 48 h ((**B**): 0 mg Se^4+^/L, (**D**): 2 mg Se^4+^/L, (**F**): 10 mg Se^4+^/L, (**H**): 20 mg Se^4+^/L, (**J**): 30 mg Se^4+^/L, (**L**): 40 mg Se^4+^/L).

The results obtained for *R. glutinis* indicate a very high amino acid quality of the protein in most conditions tested. CS values exceeded 100% in almost all cases, and many significantly exceeded the WHO/FAO reference standard. The highest CS (Figure 5) values were recorded for the sum of phenylalanine and tyrosine, reaching 259.40% in the culture with a 10 mg/L selenium supplement for 24 h and as high as 281.32% in conditions with 20 mg/L selenium for 48 h. High CS index values were also obtained for threonine, histidine, and lysine. Threonine reached over 230% in the culture conditions with 10 mg/L selenium supplement for 24 h, and a very similar level was also recorded at 20 mg Se^4+^/L after 48 h. Histidine was characterized by the highest value among all analyzed amino acids, exceeding 260% in the culture with 2 mg/L of selenium for 48 h. In turn, lysine, despite being highly sensitive in *S. cerevisiae*, remained at a high level in *R. glutinis*, reaching a maximum of over 216% at 20 mg/L of selenium after 24 h and over 205% at 2 mg Se^4+^/L after 48 h. CS values for leucine ranged from 101.51% (control culture without selenium addition after 48 h) to 175% (20 mg/L of selenium after 48 h), indicating that even under the most unfavorable conditions, they did not fall below the standard level. For isoleucine and valine, values significantly exceeding 100% were also recorded, for example, isoleucine reached 180.3% at 10 mg/L of selenium after 48 h, and valine reached 206.6% at 2 mg/L of selenium after 48 h.

**Figure 6:**
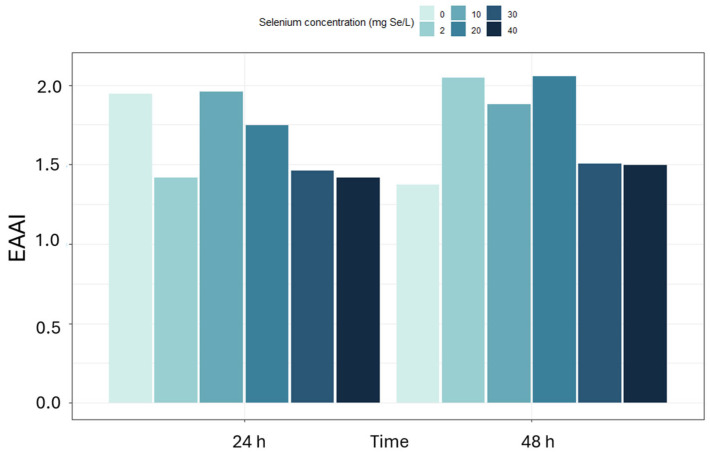
Essential amino acid index (adjusted EAAI) for *R. glutinis* yeast cultivated with selenium addition (0–40 mg Se^4+^/L) for 24 and 48 h.

The adjusted EAAI for *S. cerevisiae*, initially ranging from 0.98 to 1.71, falls below 1.0 at 40 mg Se^4+^/L. At the same time, *R. glutinis* maintains consistently high values (1.37–2.05) and even at the highest selenium doses retains an adjusted EAAI of 1.42–1.50. Moreover, the Chemical Score for phenylalanine + tyrosine reaches 281% in *R. glutinis* compared to a maximum of 223% in *S. cerevisiae*, indicating superior protein quality. EAAI index (Figure 6) also confirmed the excellent quality of the protein produced by *R. glutinis*. The highest values of this index, amounting to 2.05, were obtained after 48 h of cultivation in the presence of 2 and 20 mg Se^4+^/L. High adjusted EAAI for *S. cerevisiae*, initially ranging from 0.98 to 1.71, falls below 1.0 at 40 mg Se^4+^/L. At the same time, *R. glutinis* maintains consistently high values (1.37–2.05) and even at the highest selenium doses retains an adjusted EAAI of 1.42–1.50. Moreover, the Chemical Score for phenylalanine + tyrosine reaches 281% in *R. glutinis* compared to a maximum of 223% in *S. cerevisiae*, indicating superior protein quality. EAAI levels, exceeding 1.8, were also observed at 10 mg/L of selenium after 24 and 48 h of cultivation, as well as control conditions without adding the element. Importantly, even at the highest selenium concentration, i.e., 40 mg Se^4+^/L, the index value remained stable between 1.42 and 1.50, indicating a good balance of essential amino acids in the protein.

The *R. glutinis* strain, compared to *S. cerevisiae*, shows a much greater stability of the amino acid profile in the presence of selenium in the culture medium. The higher stability of the amino acid profile in *R. glutinis* compared to *S. cerevisiae* under selenium stress may be attributed to intrinsic metabolic traits of this non-conventional yeast. Intensive carotenoid biosynthesis, with its antioxidant activity [35], and the ability to accumulate lipids and utilize alternative amino acid metabolic pathways [27] could buffer oxidative stress and maintain protein quality. In addition, differences in selenium assimilation pathways between conventional and non-conventional yeasts may further contribute to the observed resilience, although this requires further investigation.

In the case of *S. cerevisiae*, an apparent deterioration in protein quality was observed in cultures supplemented with of 10 mg/L selenium for 48 h, where the adjusted EAAI was only 1.05, and the CS values for leucine and lysine decreased to 62.1% and 48.9%, respectively. For comparison, *R. glutinis* achieved an adjusted EAAI of 1.88 in analogous conditions, and the CS values for leucine and lysine were 160.9% and 148.3%, respectively.

This partially explains why, in our studies, decreases in the content of essential amino acids were not always proportional to the degree of oxidative stress; they could also result from gene expression regulation, not only from their direct consumption. In summary, the literature data confirm that selenium affects amino acid metabolism through oxidative stress, changes in gene expression, inhibition of enzyme activity, and redistribution of metabolites towards the synthesis of protective proteins. The results obtained for *S. cerevisiae* ATCC 7090 and *R. glutinis* CCY 20-2-26 strains are consistent with these observations, particularly in the context of reduced protein parameters (CS, EAAI) and decreased amino acid content at higher selenium doses and longer culture times.

The EAAI index also confirmed the excellent quality of the protein produced by *R. glutinis*. The highest values of this index, amounting to 2.05, were obtained after 48 h of cultivation in the presence of 2 and 20 mg Se^4+^/L. High adjusted EAAI levels, exceeding 1.8, were also observed at 10 mg/L of selenium—after 24 and 48 h of cultivation—and also in control conditions without adding the element. Importantly, even at the highest selenium concentration, i.e., 40 mg Se^4+^/L, the index value remained stable between 1.42 and 1.50, indicating a good balance of essential amino acids in the protein. The *R. glutinis* strain, compared to *S. cerevisiae*, shows a much greater stability of the amino acid profile to the presence of selenium in the culture medium. Compared with traditional protein sources such as soy and casein, selenium-enriched yeast proteins exhibited comparable or higher amino acid quality indices under specific conditions. Based on calculations using the [36,37] adult amino acid reference pattern, casein protein showed CS—150% and EAAI—2.01, while soy protein isolate showed CS—94.9% and EAAI—1.53. In our study, *Rhodotorula glutinis* maintained adjusted EAAI values ranging from 1.42 to 2.05 even under selenium stress, whereas *Saccharomyces cerevisiae* reached values up to 1.71 under control and moderately supplemented conditions. All CS and adjusted EAAI values were computed using the same [36,37] reference amino acid scoring pattern, ensuring full comparability between yeast and reference protein sources. These results suggest that selenium-enriched yeast biomass can provide protein quality equal to or exceeding that of conventional proteins such as soy and casein, highlighting its potential use in functional foods and dietary supplements.

In the case of *S. cerevisiae*, an apparent deterioration in protein quality was observed in cultures supplemented with 10 mg/L selenium for 48 h, where the adjusted EAAI was only 1.05, and the CS values for leucine and lysine decreased to 62.1% and 48.9%, respectively. Moreover, in *S. cerevisiae*, at a concentration of 40 mg Se^4+^/L for 24 h, the EAAI index dropped below 1.00 (0.98), indicating a deterioration of the amino acid balance and a limiting amino acid. Meanwhile, in the case of *R. glutinis*, under the same conditions, the EAAI remained high—1.42. Lysine and leucine remained above 100% CS in this strain, indicating the stability of protein metabolism even under oxidative stress.

In summary, *R. glutinis* demonstrated an exceptional ability to maintain high protein quality in the presence of selenium. Both the Chemical Score and EAAI values indicate that this strain may be more resistant to environmental stress than *S. cerevisiae* also while at the same time being a stable and prosperous source of well-balanced essential amino acids, regardless of cultivation time and supplementation dose.

## 3. Materials and Methods

### 3.1. Biological Material

The study used two yeast strains: *Saccharomyces cerevisiae* ATCC 7090 obtained from the American Type Culture Collection (ATCC, Manassas, VA, USA) and *Rhodotorula glutinis* CCY 20-2-26 obtained from the Yeast Culture Collection (CCY, Bratislava, Slovakia). They were stored at 4 °C on a solid YPD medium (BTL, Łódź, Poland).

### 3.2. Chemicals and Reagents

Glucose, peptone, and yeast extract were purchased from POCH S.A. (Gliwice, Poland). Sodium selenite (Na_2_SeO_3_) was obtained from Sigma-Aldrich (Steinheim, Germany). Hydrochloric acid and formic acid were obtained from POCH S.A. (Gliwice, Poland) and Chempur (Piekary Śląskie, Poland), respectively. Hydrogen peroxide was purchased from Chempur (Piekary Śląskie, Poland). Buffers for amino acid analysis were supplied by INGOS (Prague, Czech Republic), and amino acid standards were obtained from Merck/Sigma-Aldrich (Steinheim, Germany).

### 3.3. Equipment

The following equipment was used: autoclave (Systec D-45, De Ville, Raszyn, Poland), orbital shaker (Innova 44, Eppendorf, Hamburg, Germany), centrifuge (Eppendorf 5810, Hamburg, Germany), dryer (SML Zalmed, Warsaw, Poland), and amino acid analyzer (AAA-500, INGOS, Prague, Czech Republic). Filtration was carried out using Whatman 3 filter paper (GE Healthcare, Chalfont St. Giles, UK). Statistical analyses were performed using R software (v.4.1.1 Foundation for Statistical Computing, Vienna, Austria).

### 3.4. Preparation of Culture Media

A liquid YPD medium containing 2% glucose, 2% peptone, and 1% yeast extract, with a pH of 4.8, was used to prepare the yeast inoculum. To assess the ability of yeast to accumulate selenium, an experimental variant of this medium was developed, the initial pH of which was 4.8, enriched with an aqueous solution of sodium selenite (Na_2_SeO_3_). The selenium solution was prepared by dissolving 0.219 g Na_2_SeO_3_ in 100 mL of deionized water to obtain a final concentration of 1000 mg Se^4+^/L. Before adding the solution to the YPD medium, the medium and sodium selenite solution were sterilized separately in an autoclave at 121 °C for 20 min. The selenium solution was stable after the sterilization process.

### 3.5. Yeast Cultivation and Biomass Preparation

A sterile Na_2_SeO_3_ solution was added to the sterile YPD medium to obtain final selenium concentrations ranging from 1 to 40 mg Se^4+^/L. Yeast cell growth was measured using a hemocytometer in which the initial yeast cell concentration was recorded. Then, a yeast inoculum at a density of 2.3 × 10^6^ CFU/mL was added to the medium. The cultures were grown for 24 and 48 h in 500 mL flasks containing 90 mL of YPD medium for the control or YPD medium enriched with selenium. The incubation was carried out on an orbital shaker at 150 rpm and 28 °C. After cultivation, the yeast biomass was centrifuged (1530× *g*; 10 min) in 50 mL falcons and then dried (80 °C) to constant weight.

### 3.6. Amino Acid Determination

The amino acid content was determined using an AAA-500 chromatographic amino acid analyzer based on ion-exchange chromatography with post-column ninhydrin derivatization. Detection was performed at 440 nm (proline) and 570 nm (other amino acids). For the determination of total amino acids, dried yeast biomass was hydrolyzed in 6 M HCl (60 mL) at 110 °C for 23 h under nitrogen. After cooling, the hydrolysates were filtered (Whatman 3 paper) into volumetric flasks (100 mL) with demineralized water. Hydrochloric acid was then evaporated under reduced pressure (35 mbar, 60 °C), and the dry residues were dissolved in buffer at pH 2.6.

Chromatographic separation was carried out on a 250 mm Poly 8 INGOS cation-exchange column using buffers at pH 2.6, 3.0, 4.25, and 7.9. The column temperature was maintained between 55 and 74 °C, and the reactor temperature was 121 °C.

Sulfur-containing amino acids (methionine and cysteine) were determined after oxidative hydrolysis. Biomass samples were incubated with 2.5 mL of a formic acid/hydrogen peroxide mixture (9:1, *v*/*v*) at 4 °C for 16 h. The reaction was stopped by adding 0.5 mL of concentrated HCl, followed by hydrolysis in 6 M HCl (40 mL) at 125 °C for 23 h. After cooling, hydrolysates were filtered, evaporated under reduced pressure, and dissolved in citric acid buffer at pH 2.6. Chromatographic analysis was performed using buffers at pH 2.6 and 3.0, with the column temperature set at 58 °C and the reactor temperature at 121 °C.

Calculations concerning external standards were performed using the Clarity program. The chemical score (CS) and the adjusted Essential Amino Acid Index (EEAI) as the nutritional value of the protein were calculated (1):

(1) $$CS\%=\left(\mathrm{aa}/\mathrm{AA}\right)\cdot 100$$

where aa is the amino acids in the protein (mg/g) with the same amino acid content in the reference protein (AA)

(2) $$EAAI=\sqrt[{n}]{\left(aa1/AA1)\cdot (aa2/AA2)\ldots (aan/AAn\right)}$$

where EAAI (2) is calculated as the *n* root of the ratio of each essential amino acid’s content in the sample (denoted as aa) to the content of the same amino acid in the reference protein (denoted as AA), and *n* represents the total number of essential amino acids being evaluated. It should be noted that methionine and tryptophan were not quantified due to methodological limitations associated with classical acid hydrolysis. Methionine determination requires prior oxidation to methionine sulfone, whereas tryptophan degrades under acidic conditions and must be analyzed using separate alkaline hydrolysis or dedicated chromatographic methods. Therefore, the calculated adjusted EAAI values can be interpreted as comparative indicators of protein quality.

### 3.7. Statistical Analysis

The results obtained in this study were subjected to statistical analysis. All Calculations and graphical representations, including bar charts, heatmaps with hierarchical clustering, and correlation matrices, were generated using the R software (version 4.1.1). For the heatmaps, hierarchical clustering was performed using Euclidean distance as the dissimilarity metric and Ward’s method as the linkage criterion. Pearson correlation coefficients were also calculated in R. All data are expressed as mean ± standard deviation (SD) of three independent biological replicates (*n* = 3). The effects of selenium concentration, cultivation time, and yeast species on amino acid composition and protein quality indices (CS and adjusted EAAI) were evaluated using ANOVA. When significant differences were detected, Tukey’s HSD post hoc test was applied to compare means. Statistical significance was accepted at *p* < 0.05.

## 4. Conclusions

The conducted studies enabled a detailed assessment of the effect of different selenium concentrations (0–40 mg Se^4+^/L) and culture times (24 and 48 h) on the amino acid profile and biological value of protein in two yeast strains: *S. cerevisiae* ATCC 7090 and *R. glutinis* CCY 20-2-26. The results showed that both the selenium concentration and the culture time significantly modulated the content of individual amino acids and protein quality parameters. In *S. cerevisiae* ATCC 7090, prolonged cultivation and higher selenium doses resulted in a general decrease in amino acid content and protein quality. *R. glutinis* CCY 20-2-26 showed greater resistance to changes induced by the presence of selenium. In conditions of moderate supplementation (2–10 mg Se^4+^/L), an increase in the content of most amino acids (glutamic acid, glycine, arginine, alanine) was observed, while higher concentrations caused their gradual decrease. The time of cultivation affected the changes in the amino acid composition to a lesser extent than in *S. cerevisiae*. In *R. glutinis*, positive (e.g., for cysteine and histidine) and negative correlations were found, indicating more complex adaptive mechanisms. Assessment of the biological value of protein based on CS and adjusted EAAI indices showed that *R. glutinis* was characterized by higher nutritional values regardless of the culture conditions. In *S. cerevisiae*, the adjusted EAAI value ranged from 0.98 to 1.71, with a decrease in lysine and leucine CS below 100% at 10 mg Se^4+^/L and 48 h of culture, indicating their potential role as limiting amino acids. It is worth noting, however, that adjusted EAAI was presented without methionine and tryptophan. Therefore, it cannot be equivalent to the standard EAAI. The results confirm that *R. glutinis* CCY 20-2-26 shows greater metabolic resistance and the ability to synthesize well-balanced protein under selenium-induced stress compared to *S. cerevisiae* ATCC 7090. Moderate doses of selenium (2–10 mg Se^4+^/L) benefit the amino acid profile and protein quality in both strains, with these effects being more pronounced in the case of *R. glutinis*.

The obtained data may constitute a basis for further studies on the biotechnological use of selenium-enriched yeast in producing functional foods and dietary supplements. A limitation of this study is the lack of direct analyses of molecular mechanisms, such as gene expression or ROS levels; therefore, future research should combine amino acid profiling with molecular and proteomic approaches to provide a more comprehensive explanation of the observed phenomena.

## Figures and Tables

**Table 1:** Amino acid content in *S. cerevisiae* yeast after 24 and 48 h of cultivation.

Amino acid	Cultivation Time (h)											
	24						48					
	Selenium Concent in Medium (mg/L)						Selenium Concent in Medium (mg/L)					
	0	2	10	20	30	40	0	2	10	20	30	40
Ala	32.5 ± 4.6 ^cde^	25.3 ± 2.1 ^abc^	38.5 ± 0.5 ^e^	34.5 ± 2.4 ^de^	31.4 ± 3.5 ^b–e^	32.4 ± 4.0 ^cde^	25.9 ± 3.0 ^abc^	23.3 ± 1.0 ^ab^	22.3 ± 1.1 ^a^	31.1 ± 5.8 ^b–e^	29.5 ± 0.7 ^a–d^	37.6 ± 0.5 ^de^
Arg	32.8 ± 4.6 ^ab^	30.6 ± 2.1 ^a^	52.9 ± 0.5 ^c^	38.6 ± 1.7 ^b^	36.0 ± 2.6 ^ab^	29.3 ± 2.6 ^a^	30.9 ± 3.0 ^a^	29.9 ± 1.0 ^a^	30.7 ± 0.8 ^a^	33.9 ± 5.8 ^ab^	34.3 ± 0.7 ^ab^	32.9 ± 0.5 ^ab^
Asp	62.2 ± 5.3 ^c^	44.8 ± 2.8 ^b^	60.2 ± 2.2 ^c^	46.6 ± 1.2 ^b^	44.2 ± 2.2 ^b^	34.8 ± 5.3 ^a^	43.6 ± 5.8 ^b^	42.6 ± 3.0 ^ab^	41.1 ± 2.2 ^ab^	40.9 ± 1.2 ^ab^	43.6 ± 1.1 ^b^	39.9 ± 2.2 ^ab^
Cys	4.3 ± 0.1 ^abc^	4.0 ± 0.2 ^ab^	3.6 ± 0.1 ^a^	5.1 ± 0.2 ^de^	4.7 ± 0.3 ^bcd^	3.6 ± 0.4 ^a^	4.2 ± 0.1 ^abc^	3.9 ± 0.4 ^a^	4.8 ± 0.4 ^cd^	4.9 ± 0.3 ^cde^	5.5 ± 0.1 ^de^	5.7 ± 0.2 ^e^
Glu	73.6 ± 6.8 ^d^	62.4 ± 5.1 ^c^	93.7 ± 0.6 ^e^	54.5 ± 1.8 ^bc^	59.2 ± 3.7 ^c^	42.8 ± 5.1 ^a^	53.8 ± 3.1 ^bc^	58.1 ± 0.8 ^bc^	56.1 ± 2.8 ^bc^	53.3 ± 4.4 ^bc^	61.7 ± 0.8 ^c^	48.1 ± 0.6 ^ab^
Gly	26.8 ± 4.0 ^ab^	21.3 ± 1.1 ^a^	40.6 ± 0.6 ^c^	32.1 ± 2.3 ^b^	25.2 ± 6.6 ^ab^	24.5 ± 2.8 ^a^	23.1 ± 1.8 ^a^	19.6 ± 1.9 ^a^	19.9 ± 0.7 ^a^	23.3 ± 3.8 ^a^	21.4 ± 0.5 ^a^	27.4 ± 0.7 ^ab^
His	16.3 ± 1.7 ^d–f^	13.6 ± 0.8 ^a–d^	17.8 ± 0.6 ^e^	16.7 ± 1.1 ^ef^	16.1 ± 0.7 ^c–f^	10.7 ± 1.3 ^a^	11.9 ± 0.4 ^ab^	13.3 ± 0.9 ^abc^	13.4 ± 0.8 ^a–d^	14.0 ± 1.7 ^b–e^	15.7 ± 0.5 ^c–f^	12.3 ± 0.4 ^ab^
Ile	31.3 ± 4.7 ^cd^	23.7 ± 1.1 ^ab^	32.3 ± 0.8 ^d^	23.2 ± 0.9 ^ab^	24.3 ± 1.0 ^ab^	18.4 ± 1.2 ^a^	20.5 ± 3.2 ^ab^	22.5 ± 1.2 ^ab^	22.2 ± 0.2 ^ab^	24.6 ± 4.4 ^ab^	25.0 ± 0.6 ^bc^	19.9 ± 0.5 ^ab^
Leu	43.5 ± 5.5 ^b^	33.0 ± 1.2 ^a^	43.3 ± 0.6 ^b^	34.1 ± 1.6 ^a^	34.5 ± 0.9 ^a^	30.3 ± 3.6 ^a^	30.8 ± 1.4 ^a^	30.9 ± 1.3 ^a^	29.7 ± 0.7 ^a^	33.9 ± 0.4 ^a^	34.4 ± 0.7 ^a^	34.5 ± 1.0 ^a^
Lys	44.4 ± 6.5 ^b–f^	37.9 ± 2.9 ^a–e^	53.3 ± 0.9 ^f^	47.0 ± 1.1 ^c–f^	49.5 ± 1.4 ^def^	31.5 ± 3.6 ^a^	32.0 ± 4.4 ^a^	37.2 ± 3.5 ^abc^	37.4 ± 0.6 ^a–d^	45.9 ± 10.1 ^b–f^	50.0 ± 0.8 ^ef^	34.5 ± 0.6 ^ab^
Phe	23.5 ± 2.2 ^d^	20.1 ± 1.0 ^a–d^	21.4 ± 0.5 ^cd^	21.0 ± 0.6 ^cd^	20.9 ± 0.5 ^bcd^	17.6 ± 2.3 ^ab^	18.4 ± 2.0 ^abc^	19.0 ± 0.9 ^a–d^	18.6 ± 0.5 ^abc^	15.9 ± 2.2 ^a^	20.3 ± 0.7 ^a–d^	19.7 ± 0.5 ^a–d^
Pro	20.6 ± 3.7 ^a^	18.0 ± 1.2 ^a^	27.7 ± 0.5 ^b^	18.3 ± 0.7 ^a^	17.2 ± 1.2 ^a^	17.0 ± 1.4 ^a^	16.9 ± 1.3 ^a^	16.6 ± 1.7 ^a^	17.3 ± 1.1 ^a^	17.7 ± 3.7 ^a^	16.6 ± 0.5 ^a^	20.3 ± 1.9 ^a^
Ser	25.5 ± 1.6 ^ef^	20.0 ± 0.7 ^a–d^	27.6 ± 0.7 ^f^	22.0 ± 1.7 ^de^	21.9 ± 1.3 ^cde^	17.6 ± 2.6 ^a^	19.9 ± 1.3 ^a–d^	18.8 ± 0.8 ^abc^	18.0 ± 0.2 ^ab^	19.7 ± 0.2 ^a–d^	21.1 ± 0.6 ^bcd^	20.2 ± 0.3 ^a–d^
Thr	27.6 ± 2.9 ^c^	20.8 ± 0.8 ^b^	26.7 ± 0.8 ^c^	22.1 ± 0.8 ^b^	21.1 ± 1.7 ^b^	16.3 ± 3.0 ^a^	19.7 ± 1.3 ^ab^	20.0 ± 1.0 ^ab^	19.4 ± 0.2 ^ab^	20.6 ± 0.9 ^ab^	21.0 ± 1.2 ^b^	19.6 ± 0.3 ^ab^
Tyr	15.8 ± 2.5 ^c^	11.1 ± 0.8 ^ab^	13.8 ± 0.5 ^bc^	11.0 ± 0.7 ^ab^	11.0 ± 0.5 ^ab^	9.2 ± 1.7 ^a^	10.3 ± 1.8 ^a^	10.9 ± 0.7 ^ab^	10.9 ± 0.8 ^ab^	11.5 ± 0.4 ^ab^	11.4 ± 0.1 ^ab^	11.6 ± 0.7 ^ab^
Val	35.0 ± 0.5 ^e^	28.4 ± 1.0 ^a–d^	38.0 ± 0.5 ^e^	30.4 ± 1.2 ^bcd^	31.1 ± 0.7 ^cd^	25.2 ± 2.5 ^a^	27.3 ± 2.0 ^ab^	27.1 ± 0.5 ^ab^	27.1 ± 0.3 ^ab^	26.3 ± 0.8 ^a^	31.6 ± 0.9 ^d^	27.8 ± 0.6 ^abc^

^a–f^ means with the same letter did not differ significantly.

**Table 2:** Amino acid content in *R. glutinis* yeast after 24 and 48 h of cultivation.

Amino Acid	Cultivation Time (h)											
	24						48					
	Selenium Concent in Medium (mg/L)						Selenium Concent in Medium (mg/L)					
	0	2	10	20	30	40	0	2	10	20	30	40
Ala	53.4 ± 4.3 ^efg^	38.7 ± 1.4 ^ab^	58.5 ± 0.2 ^g^	40.5 ± 2.1 ^abc^	48.4 ± 0.7 ^c–f^	47.0 ± 3.3 ^cde^	35.4 ± 3.7 ^a^	51.3 ± 0.1 ^efg^	54.1 ± 2.8 ^fg^	59.0 ± 0.2 ^g^	43.1 ± 1.3 ^bc^	44.6 ± 0.5 ^bcd^
Arg	63.4 ± 11.7 ^d^	47.8 ± 1.5 ^a–d^	54.2 ± 2.1 ^cd^	49.4 ± 2.5 ^a–d^	43.4 ± 1.2 ^abc^	46.8 ± 3.3 ^a–d^	45.1 ± 8.9 ^abc^	53.8 ± 0.6 ^cd^	46.7 ± 3.3 ^a–d^	52.3 ± 1.3 ^cde^	36.8 ± 0.7 ^a^	39.0 ± 1.5 ^ab^
Asp	55.8 ± 4.2 ^cde^	41.8 ± 1.0 ^a^	61.5 ± 1.2 ^ef^	50.1 ± 5.9 ^bc^	52.1 ± 1.2 ^cd^	52.3 ± 1.5 ^cd^	43.2 ± 2.5 ^ab^	58.1 ± 1.0 ^def^	56.6 ± 4.1 ^c–f^	63.7 ± 0.1 ^f^	50.0 ± 0.5 ^bc^	50.7 ± 2 ^bcd^
Cys	3.6 ± 0.4 ^ab^	3.9 ± 0.3 ^ab^	3.7 ± 0.4 ^ab^	3.2 ± 0.3 ^a^	3.7 ± 0.2 ^ab^	4.9 ± 0.5 ^b^	3.8 ± 0.1 ^ab^	3.8 ± 0.5 ^ab^	4.2 ± 0.2 ^ab^	3.6 ± 0.9 ^ab^	4.4 ± 0.3 ^ab^	4.2 ± 0.6 ^ab^
Glu	77.7 ± 6.2 ^cd^	59.4 ± 1.8 ^a^	79.7 ± 0.6 ^d^	73.6 ± 8.2 ^bcd^	63.1 ± 0.3 ^ab^	59.6 ± 4.4 ^ab^	66.1 ± 4.1 ^abc^	102.8 ± 0.8 ^e^	76.6 ± 3.7 ^cd^	83.0 ± 0.3 ^d^	63.1 ± 0.6 ^ab^	64.0 ± 0.8 ^ab^
Gly	46.5 ± 4.8 ^de^	33.7 ± 0.8 ^b^	57.7 ± 0.1 ^f^	40.5 ± 1.5 ^cd^	36.0 ± 0.5 ^bc^	37.4 ± 2.4 ^bc^	27.8 ± 0.3 ^a^	49.5 ± 0.5 ^e^	59.7 ± 3.1 ^fg^	64.5 ± 0.6 ^g^	47.2 ± 1.0 ^e^	45.8 ± 0.6 ^de^
His	18.0 ± 1.4 ^d^	12.9 ± 0.4 ^a^	18.1 ± 0.3 ^d^	16.4 ± 2.4 ^bcd^	16.2 ± 0.7 ^bcd^	16.6 ± 1.4 ^cd^	13.8 ± 0.7 ^abc^	21.2 ± 0.0 ^e^	17.1 ± 0.9 ^d^	18.4 ± 0.1 ^de^	13.2 ± 0.2 ^a^	13.5 ± 0.5 ^ab^
Ile	28.6 ± 2.3 ^ef^	21.7 ± 0.4 ^ab^	31.3 ± 0.3 ^fg^	26.6 ± 3.6 ^cd^	25.4 ± 0.5 ^bcd^	24.0 ± 0.9 ^bc^	19.7 ± 1.2 ^b^	26.4 ± 0.3 ^cd^	30.9 ± 1.5 ^ef^	34.9 ± 0.6 ^g^	26.5 ± 0.1 ^cd^	26.9 ± 0.2 ^cde^
Leu	48.4 ± 5.2 ^cb^	36.5 ± 0.9 ^ab^	56.3 ± 1.7 ^e^	44.0 ± 4.4 ^c^	43.2 ± 0.9 ^bc^	42.3 ± 1.8 ^bc^	32.1 ± 0.7 ^a^	48.2 ± 0.1 ^cd^	54.1 ± 2.9 ^de^	60.9 ± 0.8 ^e^	45.9 ± 1.0 ^c^	46.6 ± 0.3 ^c^
Lys	51.0 ± 4.5 ^fg^	37.0 ± 1.1 ^abc^	42.8 ± 0.3 ^bcd^	53.3 ± 4.2 ^g^	44.8 ± 1.0 ^def^	41.0 ± 3.1 ^bcd^	31.7 ± 2.3 ^a^	49.7 ± 0.2 ^efg^	38.1 ± 2.1 ^a–d^	44.1 ± 0.5 ^c–f^	32.6 ± 0.5 ^a^	35.7 ± 0.7 ^ab^
Phe	19.9 ± 2.3 ^bc^	17.2 ± 1.6 ^a^	27.9 ± 0.5 ^cd^	16.6 ± 1.9 ^a^	24.5 ± 0.6 ^bc^	24.1 ± 2.3 ^cd^	16 ± 3.5 ^a^	24.2 ± 2.6 ^cd^	27.1 ± 1.4 ^cd^	33.0 ± 2.7 ^d^	29.0 ± 2.6 ^cd^	25.8 ± 0.9 ^bc^
Pro	30.9 ± 2.7 ^bcd^	24.2 ± 1.6 ^ab^	38.6 ± 2.2 ^de^	26.9 ± 3.3 ^abc^	26.1 ± 0.8 ^abc^	29.9 ± 0.5 ^a–d^	21.3 ± 2.0 ^a^	32.7 ± 2.4 ^bcd^	38.7 ± 5.6 ^de^	43.9 ± 3.8 ^e^	33.6 ± 5.5 ^cd^	33.9 ± 0.8 ^cd^
Ser	30.0 ± 2.3 ^de^	22.9 ± 0.9 ^ab^	33.2 ± 0.2 ^e^	27.9 ± 3.6 ^cd^	27.0 ± 0.5 ^bcd^	27.0 ± 1.7 ^bcd^	21.9 ± 0.6 ^a^	29.8 ± 0.2 ^de^	31.0 ± 1.4 ^de^	33.5 ± 0.5 ^e^	25.1 ± 0.4 ^abc^	26.8 ± 0.7 ^bcd^
Thr	27.4 ± 1.9 ^cde^	20.8 ± 0.8 ^a^	31.2 ± 0.1 ^e^	27.1 ± 3.6 ^cde^	27.0 ± 0.5 ^cd^	25.7 ± 1.0 ^bc^	21.5 ± 2.0 ^ab^	27.0 ± 0.6 ^cde^	27.9 ± 2.5 ^cde^	31.1 ± 0.5 ^de^	24.0 ± 0.3 ^abc^	24.7 ± 1.2 ^abc^
Tyr	13.0 ± 1.7 ^bc^	8.8 ± 0.2 ^a^	17.3 ± 0.6 ^d^	14.3 ± 1.5 ^cd^	13.9 ± 0.9 ^cd^	13.9 ± 1.0 ^cd^	9.6 ± 1.1 ^ab^	15.5 ± 3.1 ^cd^	14.5 ± 1.0 ^cd^	16.8 ± 1.1 ^cd^	13.2 ± 0.6 ^bc^	14.5 ± 0.0 ^cd^
Val	37.8 ± 8.1 ^c–f^	31.2 ± 0.8 ^abc^	41.6 ± 0.1 ^def^	28.8 ± 0.5 ^ab^	30.8 ± 0.5 ^abc^	30.3 ± 0.5 ^abc^	24.2 ± 2.4 ^a^	43.2 ± 0.2 ^ef^	41.2 ± 1.4 ^def^	46.0 ± 0.5 ^f^	33.3 ± 4.3 ^bcd^	36.0 ± 0.4 ^b–e^

^a–g^ means with the same letter did not differ significantly.

## Data Availability

The datasets used and analyzed during the current study are available from the first author upon reasonable request.
